# Production of a Monoclonal Antibody for the Detection of Forchlorfenuron: Application in an Indirect Enzyme-Linked Immunosorbent Assay and Immunochromatographic Strip

**DOI:** 10.3390/bios13020239

**Published:** 2023-02-07

**Authors:** Xingmei Lei, A. M. Abd El-Aty, Lingyuan Xu, Jing Zhao, Jia Li, Song Gao, Yun Zhao, Yongxin She, Fen Jin, Jing Wang, Lufei Zheng, Maojun Jin, Bruce D. Hammock

**Affiliations:** 1 Institute of Quality Standard and Testing Technology for Agro-Products, Chinese Academy of Agricultural Sciences, Beijing 100081, China; 2 Department of Pharmacology, Faculty of Veterinary Medicine, Cairo University, Giza 12211, Egypt; 3 Department of Medical Pharmacology, Medical Faculty, Ataturk University, Erzurum 25240, Turkey; 4 Jinhua Miaozhidizhi Agricultural Technology Co., Ltd., Jinhua 321000, China; 5 Research Center of Quality Standards for Agro-Products, Ministry of Agriculture and Rural Affairs, Beijing 100081, China; 6 Department of Entomology & Nematology and the UC Davis Comprehensive Cancer Center, University of California, Davis, CA 95616, USA

**Keywords:** forchlorfenuron, monoclonal antibody, ic-ELISA, colloidal gold nanobead immunochromatographic test strip

## Abstract

In this study, a monoclonal antibody (mAb) specific to forchlorfenuron (CPPU) with high sensitivity and specificity was produced and designated (9G9). To detect CPPU in cucumber samples, an indirect enzyme-linked immunosorbent assay (ic-ELISA) and a colloidal gold nanobead immunochromatographic test strip (CGN-ICTS) were established using 9G9. The half-maximal inhibitory concentration (IC_50_) and the LOD for the developed ic-ELISA were determined to be 0.19 ng/mL and 0.04 ng/mL in the sample dilution buffer, respectively. The results indicate that the sensitivity of the antibodies prepared in this study (9G9 mAb) was higher than those reported in the previous literature. On the other hand, in order to achieve rapid and accurate detection of CPPU, CGN-ICTS is indispensable. The IC_50_ and the LOD for the CGN-ICTS were determined to be 27 ng/mL and 6.1 ng/mL. The average recoveries of the CGN-ICTS ranged from 68 to 82%. The CGN-ICTS and ic-ELISA quantitative results were all confirmed by liquid chromatography—tandem mass spectrometry (LC-MS/MS) with 84–92% recoveries, which indicated the methods developed herein are appropriate for detecting CPPU in cucumber. The CGN-ICTS method is capable of both qualitative and semiquantitative analysis of CPPU, which makes it a suitable alternative complex instrument method for on-site detection of CPPU in cucumber samples since it does not require specialized equipment.

## 1. Introduction

Forchlorfenuron (CPPU) is a plant growth regulator widely used in agriculture. It is a cytokinin that promotes cell division and growth. When applied to plants before harvesting, it can promote tissue growth, cell mitosis and enlargement, bud development and fruit enlargement, prevent flower and fruit drop, and improve yield. After harvesting, CPPU can also be used to control a variety of yellowing leaves and diseases of fruits and vegetables. It helps to keep fruits and vegetables fresh after picking by slowing down the aging process, which can prolong the shelf life of the produce [1]. CPPU is commonly used on crops, such as cucumbers, kiwifruits, grapes, and apples, among others [2]. It is usually applied as a spray, but it can also be applied as a soil drench or as a foliar application. However, excessive use of CPPU can harm the environment and human health. Its residues in agricultural products can cause health problems, and its overuse can lead to the development of CPPU-resistant pests and diseases. Therefore, it is crucial to use CPPU in an appropriate and controlled manner and to monitor its residues in agricultural products to ensure food safety and environmental health. According to the U.S. Environmental Protection Agency (EPA), prolonged exposure to CPPU in humans can lead to adverse health effects. The EPA states that exposure to high levels of CPPU can lead to the disruption of the endocrine system. It is also toxic to nerves [3]. Studies have shown that CPPU can cause damage to heart muscle cells, leading to contractile dysfunction. Additionally, it has been shown to have an estrogenic effect, which could disrupt the endocrine system and negatively affect reproduction and development. For instance, Gong et al. [4] found that CPPU can cause cardiotoxicity in zebrafish. Specifically, they found that exposure to CPPU led to a deformity in the shape of the heart and a disruption of systolic function, in addition to anemia. Moreover, Zhang et al. [5] declared that CPPU was cytotoxic to normal Chinese hamster ovary cells. In addition, Bu et al. [6] found that CPPU has potential adverse effects in the ovaries and in steroidogenesis. Furthermore, Zhu et al. [3] stated that CPPU could promote estradiol secretion and adversely affect prepubertal female rats. Furthermore, Ping et al. [7] reported that CPPU had more potent effects on juvenile rats than adult rats in a pharmacokinetic experiment. Therefore, it is speculated that CPPU is potentially harmful to adolescent health. For public health, numerous countries have stipulated maximum residue limits for CPPU. The maximum residue limit (MRL) of forchlorfenuron in cucumbers in China and the European Union is 0.01 mg/kg. To ensure food safety and human health, it is vital to establish a simple, rapid, low-cost detection method to determine its residues.

Currently, multitudinous instrumental methods are often used to detect CPPU in various matrices. They include high-performance liquid chromatography (HPLC) [8], gas chromatography–tandem mass spectrometry (GC-MS/MS) [9], liquid chromatography–tandem mass spectrometry (LC-MS/MS) [10,11,12,13], Raman spectrometry [14], and ion mobility spectrometry [15]. These methods are sensitive and accurate. However, pretreatment is cumbersome and requires expensive equipment and professional technicians. In addition, the prolonged detection cycle makes these methods unsuitable for large-scale field screening.

Due to the advantages of simple pretreatment and high throughput, antigen–antibody binding-based immunoassay methods, especially ELISA and colloidal gold nanobead immunochromatographic test strips (CGN-ICTS), are widely used as a complement to the instrumental methods for the detection of pesticide. ELISA is the main immunoassay method for the detection of CPPU. The LOD and IC_50_ of the established enzyme-linked immunosorbent assays (ELISAs) were 0.16–12.42 ng/mL and 1.04–77 ng/mL [16,17,18,19,20,21]. Colloidal gold nanobead immunochromatographic test strips (CGN-ICTS) are widely used in the food, biological, environmental, pharmaceutical, and clinical medicine fields because of their advantages of rapidity, simplicity of operation, high sensitivity, low cost, and low professional requirements for the assay personnel [22,23,24]. They are also the mainstream method in the rapid detection of different kinds of pesticides [25,26,27]. In addition, the application of colloidal gold-based immunochromatographic test strips with portable strip readers provides a simple, fast, and sensitive test system for on-site quantitative analysis of agricultural products. 

**Figure 1:**
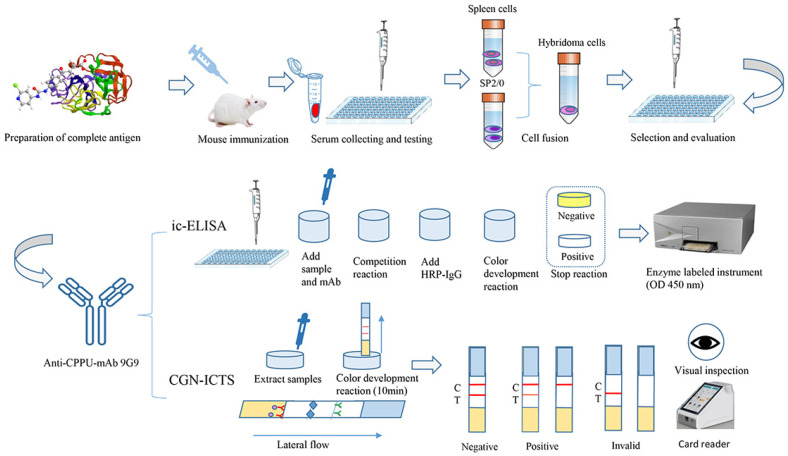
A schematic diagram for detecting CPPU.

In this study, we synthesized a semi-antigen using a previously reported CPPU semi-antigen structure and used the active ester method [20] to create a complete antigen. The hybridoma technique obtained three anti-CPPU monoclonal antibodies, and we further selected the best antibody, 9G9, by ic-ELISA. Characterizing the properties of an antibody typically includes determining its sensitivity, specificity, and any additional properties that might be relevant for its intended use. The 9G9 antibody type was IgG1. Based on this, the ic-ELISA (the IC_50_ and LOD were 0.19 ng/mL and 0.04 ng/mL, respectively) and CGN-ICTS methods (the IC_50_ and LOD were 27 ng/mL and 6.1 ng/mL, respectively) were established for detecting CPPU. Their accuracy and reliability were confirmed by LC-MS/MS. Figure 1 shows the schematic diagram for detecting CPPU using the monoclonal antibody (mAb) 9G9. The diagram illustrates the steps involved in preparing the antibody and establishing an immunoassay method for detecting CPPU.

## 2. Materials and Methods

### 2.1. Materials and Reagents

The materials and reagents are provided in Supplementary Materials S2.1.

### 2.2. Development of a Monoclonal Antibody against CPPU

#### 2.2.1. Preparation of Immunogen and Coating Antigen of CPPU

A CPPU-hapten was prepared according to a previously published method [20]. Then, the CPPU-hapten was conjugated with BSA to obtain the immunogen, and with OVA to obtain the coating antigen by the active ester method. Briefly, a mixture of 27.6 mg CPPU-hapten, 18.2 mg NHS, and 30.3 mg EDC was dissolved in 1.5 mL DMF, and then the mixture was stirred at 4 °C for 10 h. Afterward, the reaction mixture was centrifuged at 6000 rpm for 5 min, and the supernatant was used for the subsequent conjugation of the carrier proteins.

To obtain a better effect of the complete antigen, we chose different coupling ratios of hapten-proteins, including 40:1, 50:1, and 60:1, in this experiment. BSA (20 mg) and OVA (10 mg) were dissolved in 0.01 M PBS buffer (pH = 7.4). The activated hapten supernatants slowly dripped into the previously dissolved BSA and OVA solutions. The reaction was performed for four hours at room temperature with magnetic stirring. Unreacted hapten or additional minor molecules were removed by dialysis with 4 L of PBS (pH 7.4 and 0.01 mol/L) six times for 4 h each. After dialysis, the antigen was subpackaged at 1 mg/mL, snap-frozen in liquid nitrogen, and stored at −20 °C.

The prepared immunogen and coating antigen were analyzed by matrix-assisted laser desorption/ionization time-of-flight mass spectrometry (MALDI-TOF MS). The conjugation rate of hapten to BSA or OVA was measured by MALDI-TOF MS. The formula to calculate the coupling rate is as follows:

Conjugation ratio = (Mc − Mstd)/Mcppu(1)

where “Mc” represents the CPPU antigen conjugates, “Mstd” represents the BSA/OVA standard, and “Mcppu” represengts the CPPU-hapten.

#### 2.2.2. Production of Anti-CPPU mAb 

The animal experiments were conducted in strict accordance with Chinese laws and guidelines. All the animal treatment procedures were authorized by the Laboratory Animal Welfare and Ethics Committee of the Institute of Agricultural Products Quality Standards and Testing Technology, Chinese Academy of Agricultural Sciences (IQSTAP-2021-05). The monoclonal antibody (mAb) against CPPU was produced by hybridoma antibody technology [28]. The animals used in this experiment were 7-week-old pure BALB/c female mice. A brief description of the immunization protocol was as follows: the immunogen (1 mg/mL, 1 mL) was mixed with Freund’s adjuvant (1 mL) and emulsified sufficiently at 4 °C (Freund’s complete adjuvant was used for the primary immunization, and Freund’s incomplete adjuvant was used for the subsequent booster immunization). The mice were injected with the prepared immunogen at two sites, intraperitoneal and subcutaneous, for four immunization cycles. Spleen cells obtained from the final immunized mouse were fused with SP2/0 myeloma cells by polyethylene glycol. For the fusion, we chose the mouse with the highest serum titer [29]. The specific fusion operations were conducted as described elsewhere [30]. The monoclonal antibody hybridoma cell line was selected by an ic-ELISA screening procedure. Then, the hybridoma cell lines were injected into the abdominal cavity of mice to prepare as-cites antibodies, after which anti-CPPU mAb was purified by saturated ammonium sulfate [31,32], lyophilized into a powder, and stored at −20 °C.

#### 2.2.3. Evaluation of Antibody Performance

The ic-ELISA procedure was carried out according to a previously described method [30]. (1) The coating antigen diluted with coating buffer was added to a 96-well plate (100 µL per well) and then incubated at 37 °C for 3 h. (2) Fifty microliters of CPPU standard and 50 µL of anti-CPPU-mAb were added to each well after three washes with washing buffer and incubated for 0.5 h at 37 °C. (3) After three washes with washing buffer, the plates containing 100 µL HRP-labeled goat anti-mouse IgG in each well were incubated for 0.5 h at 37 °C. (4) The plate was cleaned three times with washing buffer again, and TMB chromogenic solution was added to the plate (100 µL each well). The incubation was set at 25 °C in the dark for 15 min. (5) Finally, 50 µL of the hydrochloric acid solution was pipetted into each well to stop the reaction. A microplate reader measured the absorbance value at OD 450 nm, and the data were analyzed with Origin 2021 Data Analysis Software.

In this study, we evaluated the experimental factors of the ic-ELISA, including the pH and organic solvents of the PBSTG buffer. The CPPU standards and the anti-CPPU-mAb solution were prepared in PBSTG buffer of different organic solvents (by adding methanol or acetonitrile (5%, 10%, 20%, 30%, and 40%) to the PBSTG buffer). The CPPU standards and the anti-CPPU mAb were prepared in PBSTG buffer at pH values of 5.5, 6.5, 7.5, and 8.5. The sensitivity of the anti-CPPU mAb was evaluated by the IC_50_ value, which was obtained from the standard curve. Through cross-reactivity (CR) experiments, we evaluated the specificity of the mAb. The 9G9 mAb type was identified by a mouse antibody isotyping kit from Sigma (St. Louis, MO, USA).

### 2.3. Establishment of Colloidal Gold Immunoassay

#### 2.3.1. Preparation of Antibody Probes

The pH of the colloidal gold nanobead (CGN) particles (40 nm) was adjusted to 8.0 with 0.1 mol/L potassium carbonate solution. The mAb aqueous solution was then added to the CGN solution and stirred for 10 min. A 10% (*w*/*v*) BSA solution was added to the coated CGN-mAb solution and stirred for 10 min to stabilize it, followed by centrifugation at 8500 rpm for 10 min. The supernatant was discarded, and the CGN-mAb conjugate was resolved with the colloidal gold complex solution to obtain the CGN-mAb solution.

#### 2.3.2. Preparation of Test Strips

**Figure 2:**
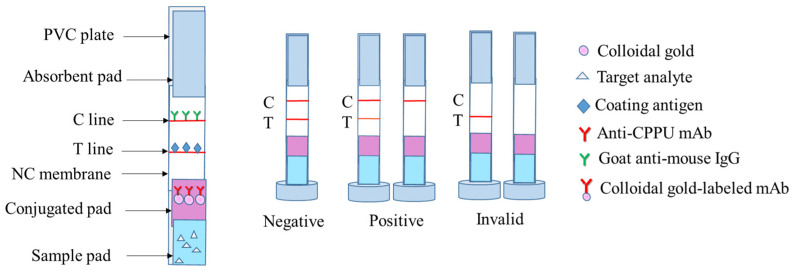
Schematic diagrams and the test principle of the CGN-ICTS.

The test strips were composed of a polyvinyl chloride (PVC) plate, sample pad, mAb-gold conjugated pad, NC membrane, and an absorbent pad. Their assembly was performed using the previous methods [33,34] (Figure 2). Goat anti-mouse IgG (Control line, C line) and CPPU-OVA (Test line, T line) were immobilized on the NC membrane at 0.6 μL/cm and separated by a distance of 0.5 cm. The CPPU-OVA had a concentration of 4 mg/mL on the T line, and the goat anti-mouse IgG had a concentration of 0.2 mg/mL on the C line. The coated NC membranes were dried at 37 °C for 0.5 h. The prepared gold standard probe solution was evenly sprayed on the gold conjugated pad and dried at 37 °C for 1 h. The sample pad, mAb-gold conjugated pad, NC membrane, and absorbent pad were manually overlapped, attached to the base plate in turn, and then cut into 4 mm width test strips. The strips were placed in a sealed bag and stored in a 4 °C and dark environment until analysis.

#### 2.3.3. Strip Performance

The sensitivity of the test strips was evaluated by analyzing forchlorfenuron standard solutions at different concentrations (1000, 500, 200, 100, 50, 20, and 10 ng/mL) in 0.02 M PB. A total of 150 µL of the standard solution was added to the test strips in the sample wells (repeated three times for each concentration). After 10 min, the T/C line was tested with the strip reader, and the Origin 2021 Data Analysis Software was used to make a linear curve.

The tolerance of the test strips to organic solvents was evaluated using different concentrations of organic solvents (methanol and acetonitrile). A total of 150 µL of PB solution containing different proportions of organic solvents was added to the test strips in the sample wells (each concentration was repeated three times). Moreover, the best organic solvent ratio was selected to prepare the standard solution (each concentration was repeated three times), the strip reader tested the intensity of the T/C line, and the linear curve was fitted by the Origin 2021 Data Analysis Software.

To evaluate the stability of the test strips, we used ten test strips to test the blank control and 50 ng/mL standard, and the strip reader read the results.

### 2.4. Detection of CPPU in Cucumber

Cucumber samples purchased from local supermarkets (Beijing, China) were homogenized and prepared for the test. After being determined to be free from CPPU with the LC-MS/MS method, blank cucumber samples were spiked with a CPPU standard solution to achieve a concentration gradient series (100, 200, 500, and 1000 ng/g). The recovery was calculated by the formula (2):

Recovery (%) = (Measured amount/Spiked amount) × 100%.(2)

#### 2.4.1. CGN-ICTS

One gram of cucumber sample was weighed and spiked with 10 mL of 10% methanol-PB (0.02 M). The supernatant was added to the CPPU standard to prepare the sample solution, with final concentrations of 10, 25, 50, 100, and 250 ng/mL. In comparison, the blank cucumber sample (1 g) was weighed and spiked with 10% methanol-PB extraction, and 150 µL of each concentration was added to the sample wells of the test strips (the experiment was repeated three times). We used the strip reader to read the reaction after 10 min and recorded the results (T/C value). The curve was fitted with the Origin 2021 Data Analysis Software (with the concentration of CPPU as the horizontal coordinate and B/B0 (the ratio of T/C at each point of the standard curve to T/C at the zero point) as the vertical coordinate). The value of T/C of the spiked sample was put into the curve to calculate the result by the software. The recovery rate was calculated by the above recovery calculation formula.

#### 2.4.2. ic-ELISA

One gram of cucumber sample was weighed, spiked with 20 mL of 10% acetonitrile-PBSTG (pH 7.5), extracted and diluted to the standard curve range. The specific assay steps were the same as in Section 2.2.3 (each concentration was repeated three times). The absorbance (OD value) was measured at 450 nm with the enzyme standard, and the curve was fitted with Origin 2021 Data Analysis Software (with concentration of CPPU as the horizontal coordinate and B/B0 (the OD value of each point of the standard curve to the OD value at zero point) as the vertical coordinate).

#### 2.4.3. LC-MS/MS

A cucumber sample (10 g) was weighed into 50 mL centrifuge tubes, to which 10 mL of acetonitrile was added, vortexed, and shaken for 3 min. Then, 4 g of anhydrous magnesium sulfate and 1 g of sodium chloride were added to the tube, vortexed for 1 min, and then centrifuged at 5000 r/min for 5 min. Subsequently, 1.5 mL of the supernatant was aspirated into a 2 mL centrifuge tube containing 50 mg of PSA and 150 mg of anhydrous magnesium sulfate, vortexed for 30 s, and centrifuged at 5000 r/min for 5 min. The supernatant was passed through an organic membrane for LC–MS/MS detection. All the analyses of CPPU were performed on a Shimadzu LCMS-8060 liquid chromatography–mass spectrometer (Shimadzu, Japan) equipped with an electrospray ionization (ESI) source. The LC–MS/MS detection conditions are shown in Table S11 in the Supplementary Materials.

## 3. Results

### 3.1. Development of a Monoclonal Antibody against CPPU

#### 3.1.1. Characterization of Immunogen and Coating Antigen of CPPU

CPPU-hapten was conjugated with a carrier protein (BSA or OVA) as an immunogen and coating antigen. The immunogen and coating antigens with different coupling ratios were prepared by the active ester method and measured by MALDI-TOF MS. The single charge ion peaks of BSA and OVA standards were 67,217.314 and 44,630.281, respectively. The molecular weight of the CPPU was 347.75. The MALDI-TOF MS analysis results of the immunogen and coating antigen are shown in Figure S1 in the Supplementary Materials. The coupling results are listed in Table 1. The results indicated that the CPPU-hapten-BSA and CPPU-hapten-OVA were successfully synthesized. According to the data and our previous experimental study, it is inferred that the immunogen and coating antigen with the higher actual coupling ratio may have a better immune effect and binding effect. Therefore, the immunogen that obtained a ratio of 50:1 was selected as the optimum immunized antigen. The coating antigen, which obtained a ratio of 60:1, was selected as the ic-ELISA coating antigen.

#### 3.1.2. Production and Selection of Anti-CPPU mAb

After the third and fourth immunizations, within 3–5 days, we collected blood samples from each mouse’s eye socket and detected the inhibition rate to select the best one. The serum titer of the fusion mouse is shown in Tables S1 and S2 in the Supplementary Materials. The monoclonal cell lines obtained by hybridoma technique fusion were cultured and injected into paraffin-treated BALB/c pure-line female mice to prepare ascites antibodies. The three cell lines with superior inhibitory effects were 9G9, 9A10, and 1B6, which were screened for ascites and purified antibodies by a checkered ic-ELISA. The results are shown in Tables S3–S5 (ascites antibody) and Tables S6–S8 (purified antibody) in the Supplementary Materials. We selected one antibody as the most suitable antibody (9G9).

#### 3.1.3. Characterization of Anti-CPPU mAb

**Figure 3:**
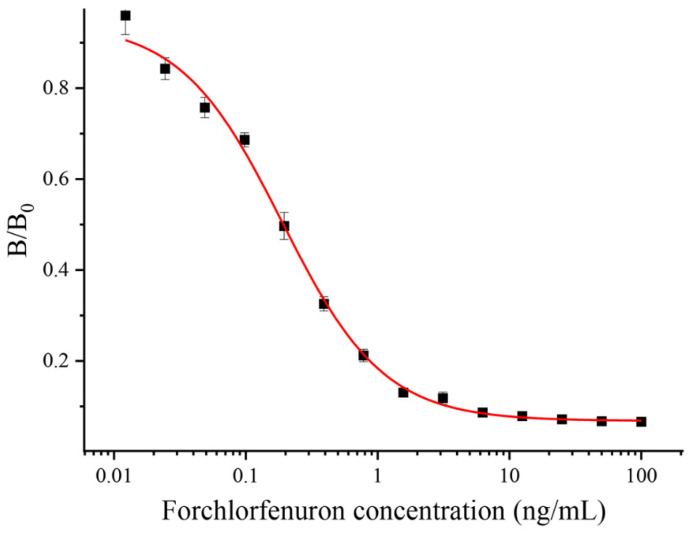
Ic-ELISA curve of the 9G9 mAb.

The sensitivity of an antibody is an essential indicator to assess its properties, and the IC_50_ value reflects the sensitivity of the antibody. This study purified and characterized antibodies generated from the 9G9 monoclonal cell line. The optimal operational concentrations of mAb and coating antigens were determined by a checkerboard indirect ELISA with 32,000 and 16,000 dilutions of 1 mg/mL, respectively. In this study, the effects of organic solvents and pH on the antibody performance were also investigated by an ic-ELISA. PBSTG solutions containing different concentrations of organic solvents (methanol and acetonitrile) and different pH values were prepared; antigen–antibody binding was determined by an ic-ELISA; and the IC_50_ values were calculated. As shown in Figure S2c and Table S10 in the Supplementary Materials, the pH effect on the antibody–antigen combination was mild, and the IC_50_ showed superior consistency under different pH conditions. The IC_50_ value was the smallest at pH 7.5. The effect of the organic solvent on the antibody–antigen combination is larger, and the IC_50_ increased after adding methanol or acetonitrile to the PBSTG. Figure S2a,b and Table S9 in the Supplementary Materials show that the minimal IC_50_ value of acetonitrile–PBSTG is 10% or less. Finally, a pH of 7.5 PBSTG (contain 10% acetonitrile) were chosen as the extraction buffer solution. The CPPU standard was diluted to 100, 50, 25, 12.5, 6.25, 3.12, 1.56, 0.78, 0.39, 0.19, 0.1, 0.05, 0.025, 0.0125, and 0 ng/mL in pH 7.5 PBSTG (0.01 M, contain 10% acetonitrile) for subsequent experiments. The standard curve for the ic-ELISA is shown in Figure 3. Each value was repeated three times independently. The IC_50_ of the anti-CPPU 9G9 mAb was 0.19 ng/mL, and the working range of the standard curve (IC_20_–IC_80_) was 0.04 to 0.87 ng/mL. The R^2^ of the standard curve was 0.996. As shown in Table 2, the sensitivity of the anti-CPPU 9G9 mAb was higher than that in the previously reported literature (same hapten structure). It has been suggested that the anti-CPPU 9G9 mAb is more suitable for establishing an extremely sensitive immunoassay.

The specificity of antibodies is one of the most significant factors affecting the detection of analytes in samples. Cross-reactivity (CR) values from six structural simulations were selected to evaluate the specificity of mAb against CPPU. The CR values were calculated by the equation [35]:

CR (%) = IC_50_ of CPPU/IC_50_ of structural analog × 100% (3)

The results are shown in Table 3. The CPPU mAb cross-reacted with thidiazuron, and the CR value was 20.2%. A probable reason for this specific binding is the bioisosterism [36] that occurs between the 2-chloropyridyl ring of CPPU and the thiadiazole ring of thidiazuron [15,37]. The CPPU mAb had no cross-reaction (CR < 0.01%) with diuron, linuron, clopyralid, or clofentezine. The anti-CPPU mAb showed excellent specificity. The 9G9 mAb type was identified as IgG1, using a mouse antibody isotyping kit from Sigma, St. Louis, MO, USA. The data are shown in Table S12 in the Supplementary Materials.

### 3.2. Establishment of a Colloidal Gold Immunoassay Method

#### 3.2.1. Optimization of the Strip Test

Antibodies rely on electrostatic interactions to bind to the surface of colloidal gold, and the pH of the solution affects their binding. A low pH value will destroy the electrostatic charge on the surface of colloidal gold, which can easily lead to colloidal gold polymerization and subsidence. A high pH value will result in insufficient force between the colloidal gold and the antibody, reducing the amount of adsorbed protein. When the pH of the solution is adjusted to be either antibody isoelectric or weakly alkaline, the binding of the antibody to colloidal gold is strong and the most stable. The results of the pH optimization are shown in Figure S3. Combining the color shift and OD value, the pH at which the color remained bright red and the OD value was stable was selected as the optimal condition, so the optimal amount of the K_2_CO_3_ addition was 7 μL.

To ensure the excellent color development effect of the test strips, different concentrations of the coated antigens and secondary antibody combination (coating antigens and secondary antibody: (1) 2 and 1 mg/mL, (2) 1.5 and 0.8 mg/mL, (3) 3 and 0.4 mg/mL, (4) 4 and 0.2 mg/mL) were prepared for testing. The test strip should be negative in PB buffer. That is, the strength of the C line is greater than that of the T line. The test results are shown in Figure S4, and the ratio of coated antigens and secondary antibodies (4 and 0.2 mg/mL) was finally selected.

#### 3.2.2. Strip Performance

**Figure 4:**
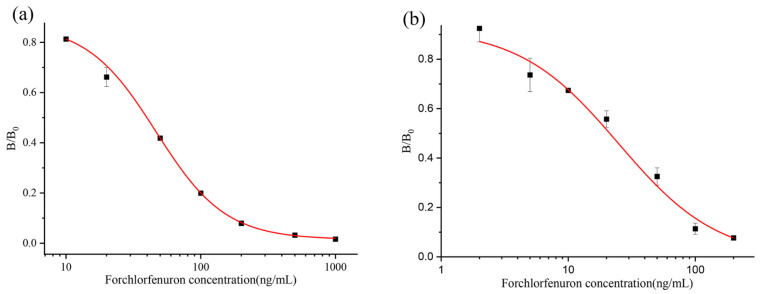
CGN-ICTS curve of CPPU in PB (**a**) 10% methanol-PB (**b**).

The effects of two organic reagents, methanol and acetonitrile, on the test strips were tested, and the results are shown in Figures S5a,b. The PB containing 1% and 10% methanol or 1% acetonitrile did not affect the color of the test strips. However, the PB of 20% and 50% methanol or 10% and 20% acetonitrile content made the color of the test strips significantly weaker. At 100% methanol, or 50% and 100% acetonitrile, the test strip was no longer laminated, and no color appeared. Therefore, in practice, the final methanol content of the substance to be measured should not be higher than 10%, and the acetonitrile content should not be higher than 1%. Then, the standard was prepared in 10% methanol-PB, the OD value was measured, and the curve was fitted using Origin 2021 Data Analysis Software, as shown in Figure 4a,b. In the PB, the IC_50_ was 39.9 ng/mL. In the 10% methanol-PB, the IC_50_ value was 21.2 ng/mL, and the detection range was 4.6–76.9 ng/mL. We chose 10% methanol-PB as the extraction buffer.

To evaluate the stability of the test strips during the test, 20 test strips were randomly selected and tested with 0 ng/mL and 50 ng/mL CPPU. The experimental results are shown in Figure S6. The RSDs results about the test of negative CK and 50 ng/mL of CPPU were 7.9% and 2.0%, respectively, which proved that these test strips could be used for the actual assay based on their superior stability.

### 3.3. Recovery

**Figure 5:**
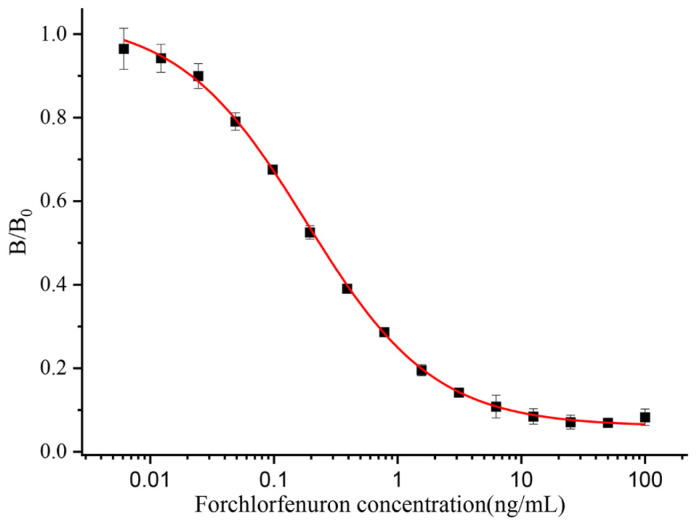
Ic-ELISA curve of CPPU in cucumber samples.

**Figure 6:**
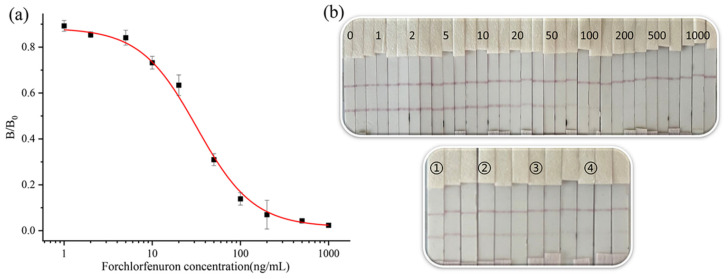
(**a**) CGN-ICTS curve of CPPU in cucumber samples. (**b**) The strip image results for CPPU (0, 1, 2, 5, 10, 20, 50, 100, 200, 500, and 1000 represent CPPU standard concentrations of 0, 1, 2, 5, 10, 20, 50, 100, 200, 500, and 1000 ng/mL, respectively). Analysis of samples by ICTS. Detection of CPPU in cucumber (①②③④ represent CPPU concentrations of 100, 200, 500, and 1000 ng/g, respectively).

To test the reliability of the developed assay in practical applications, cucumber samples were spiked with CPPU at concentrations of 0, 100, 200, 500, and 1000 ng/g. For the ic-ELISA, the IC_50_ of the standard curve was 0.23 ng/mL, and the working range of the standard curve (IC_20_–IC_80_) was 0.05 to 1.55 ng/mL (Figure 5). The average recoveries of CPPU were 70–104%, and the RSDs were 1.7–5.7% (Table 4). The standard curve is shown in Figure 5. For the CGN-ICTS, the IC_50_ of the standard was 27.0 ng/mL, and the working range of the standard curve (IC_20_–IC_80_) was 6.1 to 84.5 ng/mL (Figure 6a,b). The LOD was 6.1 ng/mL. The average recoveries of CPPU were 68–82%, and the RSDs were 3.7–7.9% (Table 4). During on-site detection, the detection results can be judged by the strip reader to interpret the T/C line strength value. For the LC-MS/MS, the average recoveries of CPPU were 84–92%, and the RSDs were 0.4–1.7% (Table 4). There was high agreement between the ic-ELISA, CGN-ICTS results, and the LC-MS/MS results. The CGN-ICTS can simultaneously be used for visual (qualitative) or strip reader (semiquantitative or quantitative) assessment. A short extraction time allowed the CGN-ICTS to be more suitable for on-site tests. The test strips can be used for qualitative testing of actual cucumber samples.

## 4. Conclusions

We prepared an anti-CPPU mAb (9G9) with high sensitivity and specificity in this study. Based on this, ic-ELISA and colloidal gold immunoassay methods were established for detecting CPPU in cucumber samples. LC-MS/MS confirmed the accuracy of these two methods.

The IC_50_ value of the ic-ELISA was 0.19 ng/mL. The LOD was 0.04 ng/mL, and the standard curve (IC_20_–IC_80_) had an operating range of 0.04–0.87 ng/mL in the assay buffer. For the detection in the cucumber samples, the sensitivity of the ic-ELISA in this study was higher than that in the previously reported literature (the IC_50_ for CPPU was 1.04 ng/mL, and the LOD was 0.16 ng/mL in the detection of cucumber samples by Xinmei Liu et al.) [16]. The cross-reactivity of the five compounds was low (CR < 0.01%), except for thidiazuron (CR = 20.2%), which has a highly similar structure. That reflects the better specificity of the anti-CPPU monoclonal antibody. The average recoveries of the ic-ELISA ranged from 70% to 104%, with RSDs between 1.7–5.7% in the cucumber samples. Moreover, the IC_50_ value of the colloidal gold immunoassay method was 27.0 ng/mL in the cucumber samples. The LOD was 6.1 ng/mL, and the detection range was 6.1–84.5 ng/mL. The recovery was 68–82%, and the RSD was 3.7–7.9%.

LC–MS/MS confirmed the colloidal gold immunoassay method and the ic-ELISA quantitative results with 84–92% recoveries. The excellent agreement between these three methods proves that the ic-ELISA and colloidal gold immunoassay methods are appropriate for detecting forchlorfenuron in cucumber. In addition, the colloidal gold immunoassay method is capable of 10–15 min on-site qualitative and semiquantitative analysis of CPPU in cucumber samples by a portable strip reader. The colloidal gold immunoassay method is simple, independent of complex instruments, and is of great value for on-site screening of CPPU residuals by unskilled personnel. Therefore, this work aims to advance the application of colloidal gold immunoassay to routine monitoring of CPPU residues in foods.

## Figures and Tables

**Table 1:** Characterization of the CPPU immunogen and coating antigen.

**(A) Characterization Results of the CPPU Immunogen.**			
**Feeding Ratio of Hapten to a Carrier** **Protein**	**Molecular Weight of** **Hapten-BSA (Da)**	**Mass Change ∆m (Da)**	**Actual Coupling Ratio of Hapten-BSA**
40:1	73,072.926	5855.61	16.84
50:1	74,068.026	6850.71	19.70
60:1	74,064.552	6847.24	19.69
**(B) Characterization Results of the CPPU Coating Antigen**			
**Feeding Ratio of Hapten to a Carrier** **Protein**	**Molecular Weight of** **Hapten-OVA (Da)**	**Mass Change ∆m (Da)**	**Actual Coupling Ratio of Hapten-OVA**
40:1	45,551.489	921.21	2.65
50:1	45,780.576	1150.30	3.31
60:1	45,829.570	1199.29	3.45

**Table 2:** Comparison with previously reported (same hapten structure) IC_50_ of anti-CPPU antibodies.

IC_50_ (ng/mL)	Reference
0.19	In this work
1.04	[16]
48	[20]

**Table 3:** Cross-reactivity (CR) of CPPU and other structural analogs.

Analytes	Chemical Structure	IC_50_ (ng/mL)	Cross-Reactivity (%)
Forchlorfenuron	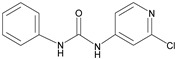	0.19	100
Thidiazuron	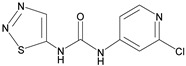	0.94	20.2
Diuron	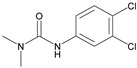	25,500	<0.01
Linuron	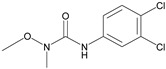	-	<0.01
Clopyralid	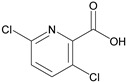	-	<0.01
Clofentezine	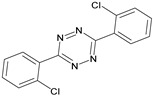	-	<0.01
Gibberellic acid	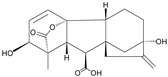	-	<0.01

Note: “-” indicates that the IC_50_ value cannot be measured.

**Table 4:** Average recoveries of CPPU in cucumber samples measured by ic-ELISA, CGN-ICTS, and LC-MS/MS.

Sample	Fortified (ng/g)	ic-ELISA			CGN-ICTS			LC-MS/MS		
		Detected (ng/g)	Recovery (%)	RSD (%)	Detected (ng/g)	Recovery (%)	RSD (%)	Detected (ng/g)	Recovery (%)	RSD (%)
cucumber	0	ND	-	-	Negative	-	-	ND	-	-
	100	72	72	5.7	68	68	7.1	85	85	1.7
	200	140	70	1.7	165	82	3.7	184	92	0.4
	500	426	85	2.4	387	77	7.9	418	84	0.5
	1000	1035	104	5.2	712	71	7.6	852	85	1.1

Note: ND indicates that CPPU is not detected in the sample.

## Data Availability

Not applicable.

## Supplementary Materials

The following supporting information can be downloaded at https://www.mdpi.com/article/10.3390/bios13020239/s1, S2.1. Materials and Reagents. Table S1. Serum potency and inhibition rate of CPPU in mice. (Third immunization); Table S2. Serum potency and inhibition rate of CPPU in mice. (Fourth immunization); Table S3. The checkered ic-ELISA result of ascites antibody 9G9; Table S4. The checkered ic-ELISA result of ascites antibody 9A10; Table S5. The checkered ic-ELISA result of ascites antibody 1B6; Table S6. The checkered ic-ELISA result of purified antibody 9G9; Table S7. The checkered ic-ELISA result of 9A10; Table S8. The checkered ic-ELISA result of 1B6; Table S9. Optimization of ic-ELISA with different organic solvent contents in PBSTG buffer. Table S10. Optimization of ic-ELISA with different pH values of PBSTG buffer; Table S11. LC-MS/MS detection conditions; Table S12. Anti-CPPU 9G9 mAb type; Figure S1. MALDI-TOF MS results of the immunogen (A) and coating antigen (B); Figure S2. Optimization of ic-ELISA with different organic solvent contents and pH values in PBSTG buffer: (a) methanol, (b) acetonitrile, and (c) pH; Figure S3. pH optimization of colloidal labeled antibody (the volume of K_2_CO_3_); Figure S4. Optimization of coating antigen and secondary antibody concentration (coating antigen/secondary antibody: (1) 2 and 1 mg/mL, (2) 1.5 and 0.8 mg/mL, (3) 3 and 0.4 mg/mL, (4) 4 and 0.2 mg/mL); Figure S5. Organic solvent content on the strip: (a) methanol, and (b) acetonitrile; Figure S6. Test stability of CGN-ICTS (a) CK (b) 50 ng/mL.
