# Supplementary material for: Production of a Monoclonal Antibody for the Detection of Forchlorfenuron: Application in an Indirect Enzyme-Linked Immunosorbent Assay and Immunochromatographic Strip
Source: Biosensors (Basel). 2023 Feb 7;13(2):239. doi: 10.3390/bios13020239 (PMC9954037; doi:10.3390/bios13020239)
Supplement: Supplementary file 1 [file biosensors-13-00239-s001.zip › biosensors-2144347-supplementary.pdf]

biosensors

MDPI

Supplementary Materials

Production of a Monoclonal Antibody for the Detection of Forchlorfenuron: Application in an Indirect Enzyme-Linked Immunosorbent Assay and Immunochromatographic Strip

Xingmei Lei, A. M. Abd El-Aty, Lingyuan Xu, Jing Zhao, Jia Li, Song Gao, Yun Zhao, Yongxin She, Fen Jin, Jing Wang, Lufei Zheng, Maojun Jin and Bruce D. Hammock

2.1. Materials and Reagents

Female Balb/c mice (7 weeks old) were supplied by SPF (Beijing) Biotechnology Co., Ltd. (Beijing, China). The mouse Sp2/0-Ag14 myeloma cell line was supplied by the Cell Resource Center of Peking Union Medical College (Beijing, China). Forchlorfenuron (CPPU), diuron, linuron, and clopyralid standards were acquired from Dr. Ehren-storfer GmbH (Augsburg, Germany). Gibberellic acid, thidiazuron, and clofentezine standards were secured from TM standard (Beijing, China). CPPU-hapten, complete and incomplete Freund's adjuvant, bovine serum albumin (BSA), ovalbumin (OVA), 1-(3-dimethylaminopropyl)-3-ethylcarbodiimide hydrochloride (EDC), N-hydroxysuccinimide (NHS), polyethylene glycol 2000 (PEG-2000), hypoxanthine and thymidine (HT) medium supplements, hypoxanthine, aminopterin and thymidine (HAT), dimethyl sulfoxide (DMSO), cell freezing medium dimethyl sulfoxide (DMSO; serum-free), horse-radish-peroxidase-labeled TMB (3,30,5,50-tetramethyl benzidine) substrate solution, and 50% (w/v) polyethylene glycol solution were provided by Sigma–Aldrich (St. Louis, MO, USA). Cell culture medium (Dulbecco's modified Eagle's medium; DMEM) and fetal bovine serum (FBS) were purchased from Gibco BRL (Pais-ley, Scotland). GIBCO® Australian Premium FBS, GIBCO®. DMEM basic (1X) basal culture medium, L-glutamine solution, and penicillin–streptomycin solution were procured from Thermo Fisher Scientific (Waltham, MA, USA). N,N-Dimethylformamide (DMF) was supplied by Tianjin Seans Biochemical Technology Co. (Tianjin, China). Horseradish peroxidase-labeled goat anti-mouse IgG (IgG-HRP) was purchased from Jackson Immune Research Laboratories Co. (West Grove, PA, USA). Goat anti-mouse IgG, methanol, acetonitrile, hydrochloric acid (HCl), sodium chloride (NaCl), sodium hydrogen phosphate dodecahydrate disodium hydrogen phosphate dodecahydrate ($Na_2HPO_4 \cdot 12H_2O$), potassium dihydrogen phosphate (KH_2PO_4), gelatin, Tween-20, sodium carbonate (Na_2CO_3), analytical-grade sodium bicarbonate ($NaHCO_3$), citric acid monohydrate ($C_6H_{10}O_8$), hydrogen peroxide (H_2O_2), and potassium carbonate (K_2CO_3) were obtained from Sinopharm Chemical Reagent Co. (Beijing, China).

Colloidal gold (0.01%, w/v) 40 nm, colloidal gold complex solution, nitrocellulose (NC) membranes (Sartorius CN120), polyvinyl chloride (PVC) plates, sample pads and ab-sorbent pads were purchased from Jieyi Biotech Co, Ltd. (Shanghai, China). Syringes (1 and 5 mL) were supplied by Shandong Zhu Pharmaceutical Co. (Shandong, China). Formic acid (HPLC), ammonium formate (HPLC), and cell pipettes (1, 5, and 10 mL) were purchased from Thermo Fisher Scientific (Waltham, MA, USA). 96-MicroWell™ transparent plates, cell culture plates (6-well™, 24-well™, and 96-well™) and centrif-ugal tubes were secured from Costar (Corning, USA). The glassware used in the experiments was strictly cleaned and sterilized before use. The tips of the guns for cell culture were wrapped with newspaper and sterilized at 121°C for 20 min after autoclave drying before use. Cucumber samples were procured from local supermarkets (Beijing, China).

Table S1. Serum potency and inhibition rate of CPPU in mice. (Third immunization).

**Dilution of immunogen** (**Serum dilution**)	**0.5×10³**			**1×10³**			**2×10³**			**4×10³**		
	**C**	**I**	**IR**	**C**	**I**	**IR**	**C**	**I**	**IR**	**C**	**I**	**IR**
2×10³	3.1575	3.4519	-9%	3.3295	3.0931	7%	3.4129	1.8489	46%	3.1061	1.0187	67%
4×10³	2.979	3.5435	-19%	3.5799	2.0973	41%	3.4803	1.28	63%	3.1077	0.7472	76%
8×10³	3.4544	2.9655	14%	3.6383	1.4285	61%	3.1615	0.8687	73%	2.035	0.3826	81%
16×10³	3.4254	1.6507	52%	2.9929	0.8022	73%	2.2554	0.4805	79%	1.2281	0.2365	81%

Note: "C" represents control wells, "I" represents inhibition wells, and "IR" represents inhibition rate. The inhibitory concentration of CPPU was 1000 ng/mL.

Table S2. Serum potency and inhibition rate of CPPU in mice. (Fourth immunization).

**Dilution of immunogen** (**Serum dilution**)	**0.5×10³**			**1×10³**			**2×10³**			**4×10³**		
	**C**	**I**	**IR**	**C**	**I**	**IR**	**C**	**I**	**IR**	**C**	**I**	**IR**
8×10³	3.3838	2.9745	12.10%	3.7764	1.6374	56.64%	3.744	0.8641	76.92%	3.0526	0.5278	82.71%
16×10³	2.7014	2.1559	20.19%	3.1686	1.0018	68.38%	3.3676	0.5876	82.55%	2.4274	0.2995	87.66%
32×10³	3.0599	1.1091	63.75%	2.859	0.5308	81.43%	2.3569	0.3232	86.29%	1.5893	0.1883	88.15%
64×10³	2.4069	0.4927	79.53%	1.8739	0.2672	85.74%	1.2435	0.1824	85.33%	0.8106	0.1226	84.88%

Note: "C" represents control wells, "I" represents inhibition wells, and "IR" represents inhibition rate. The inhibitory concentration of CPPU was 1000 ng/mL.

Table S3. The checkered ic-ELISA result of ascites antibody 9G9.

**Dilution of immunogen** (**Serum dilution**)	**2×10³**			**4×10³**			**16×10³**		
	**C**	**I**	**IR**	**C**	**I**	**IR**	**C**	**I**	**IR**
2×10³	3.4184	2.3685	30.7%	3.3464	1.667	50.2%	2.8866	0.5528	80.8%
4×10³	3.3394	1.7776	46.8%	3.2532	1.0515	67.7%	2.9336	0.3357	88.6%
8×10³	3.3392	1.1236	66.4%	3.1713	0.68	78.6%	2.8714	0.2378	91.7%
16×10³	3.2914	0.6905	79.0%	3.1735	0.4467	85.9%	2.7216	0.1722	93.7%
32×10³	3.1718	0.549	82.7%	3.0324	0.3133	89.7%	2.6577	0.1468	94.5%
64×10³	2.9532	0.3948	86.6%	2.7744	0.25	91.0%	2.3482	0.1259	94.6%
128×10³	2.6788	0.318	88.1%	2.5474	0.2238	91.2%	2.0429	0.1197	94.1%
256×10³	2.1066	0.2943	86.0%	2.103	0.2165	89.7%	1.6288	0.1222	92.5%
Dilution of immunogen (Serum dilution)	32×10³			64×10³			128×10³		
	C	I	IR	C	I	IR	C	I	IR
2×10³	2.7256	0.3205	88.2%	2.3273	0.1895	91.9%	1.9021	0.1371	92.8%
4×10³	2.5853	0.2034	92.1%	2.1799	0.1237	94.3%	1.6457	0.1025	93.8%
8×10³	2.4904	0.1422	94.3%	2.1088	0.0987	95.3%	1.5097	0.0858	94.3%
16×10³	2.3841	0.1154	95.2%	1.9032	0.0891	95.3%	1.347	0.0801	94.1%
32×10³	2.1892	0.1034	95.3%	1.6715	0.0744	95.5%	1.0796	0.076	93.0%
64×10³	1.8346	0.0954	94.8%	1.3339	0.0769	94.2%	0.8227	0.0745	90.9%
128×10³	1.5359	0.094	93.9%	1.018	0.0809	92.1%	0.6248	0.0769	87.7%
256×10³	1.1251	0.095	91.6%	0.7308	0.0813	88.9%	0.4723	0.0828	82.5%

Note: "C" represents control wells, "I" represents inhibition wells, and "IR" represents inhibition rate. The inhibitory concentration of CPPU was 100 ng/mL.

Table S4. The checkered ic-ELISA result of ascites antibody 9A10.

**Dilution of immunogen**	**2×10^{3}**			**4×10^{3}**			**16×10^{3}**		
**(Serum dilution)**	**C**	**I**	**IR**	**C**	**I**	**IR**	**C**	**I**	**IR**
2×10^{3}	1.6272	2.9662	-82.29%	2.1443	1.4481	32.47%	1.8106	0.3305	81.75%
4×10^{3}	1.6972	2.0861	-22.91%	2.8353	0.9124	67.82%	1.3893	0.2497	82.03%
8×10^{3}	1.5777	1.2474	20.94%	2.7358	0.6119	77.63%	1.1477	0.2112	81.60%
16×10^{3}	2.3325	0.9615	58.78%	2.5592	0.5275	79.39%	0.9045	0.197	78.22%
32×10^{3}	2.9709	0.6774	77.20%	1.8761	0.4195	77.64%	0.6921	0.1826	73.62%
64×10^{3}	2.2124	0.5794	73.81%	1.2721	0.3925	69.15%	0.5921	0.186	68.59%
128×10^{3}	1.6379	0.5133	68.66%	1.0824	0.3726	65.58%	0.5408	0.1776	67.16%
256×10^{3}	1.1824	0.5138	56.55%	1.0252	0.3643	64.47%	0.5123	0.1857	63.75%
Dilution of immunogen	32×10^{3}			64×10^{3}			128×10^{3}		
(Serum dilution)	C	I	IR	C	I	IR	C	I	IR
2×10^{3}	0.9169	0.1877	79.53%	0.5158	0.1354	73.75%	0.2934	0.1155	60.63%
4×10^{3}	0.7255	0.1499	79.34%	0.4364	0.1162	73.37%	0.271	0.0971	64.17%
8×10^{3}	0.606	0.1378	77.26%	0.3476	0.1064	69.39%	0.2303	0.0933	59.49%
16×10^{3}	0.535	0.1261	76.43%	0.3005	0.097	67.72%	0.1932	0.0874	54.76%
32×10^{3}	0.4288	0.1237	71.15%	0.2461	0.0884	64.08%	0.1746	0.0861	50.69%
64×10^{3}	0.3762	0.1266	66.35%	0.244	0.0954	60.90%	0.1624	0.0845	47.97%
128×10^{3}	0.385	0.1225	68.18%	0.2536	0.0957	62.26%	0.1676	0.0878	47.61%
256×10^{3}	0.3152	0.128	59.39%	0.242	0.1174	51.49%	0.172	0.0914	46.86%

Note: "C" represents control wells, "I" represents inhibition wells, and "IR" represents inhibition rate. The inhibitory concentration of CPPU was 100 ng/mL.

Table S5. The checkered ic-ELISA result of ascites antibody 1B6.

**Dilution of immunogen**	**2×10^{3}**			**4×10^{3}**			**16×10^{3}**		
**(Serum dilution)**	**C**	**I**	**IR**	**C**	**I**	**IR**	**C**	**I**	**IR**
2×10^{3}	2.3849	3.3417	-40.1%	2.659	2.0825	21.7%	3.8157	0.4519	88.2%
4×10^{3}	2.9152	2.5852	11.3%	2.8399	1.3301	53.2%	3.8489	0.3482	91.0%
8×10^{3}	3.8193	1.6591	56.6%	3.7097	0.8939	75.9%	3.4479	0.2674	92.2%
16×10^{3}	2.6946	1.2822	52.4%	3.1027	0.6541	78.9%	3.3567	0.2179	93.5%
32×10^{3}	3.4251	0.8691	74.6%	3.3407	0.5391	83.9%	2.6182	0.1957	92.5%
64×10^{3}	3.3325	0.6521	80.4%	3.3298	0.4092	87.7%	1.6793	0.1797	89.3%
128×10^{3}	2.51	0.5654	77.5%	2.3323	0.3737	84.0%	1.1787	0.1827	84.5%
256×10^{3}	2.0327	0.6321	68.9%	1.8003	0.4035	77.6%	0.9017	0.1895	79.0%
Dilution of immunogen	32×10^{3}			64×10^{3}			128×10^{3}		
(Serum dilution)	C	I	IR	C	I	IR	C	I	IR
2×10^{3}	3.2829	0.2627	92.0%	2.5512	0.1725	93.2%	1.4655	0.1286	91.2%
4×10^{3}	3.0915	0.192	93.8%	2.1095	0.1375	93.5%	1.2362	0.1139	90.8%
8×10^{3}	2.9857	0.1611	94.6%	1.7683	0.1107	93.7%	0.9349	0.1033	89.0%
16×10^{3}	2.2663	0.1484	93.5%	1.2486	0.1066	91.5%	0.6988	0.0898	87.1%
32×10^{3}	1.6491	0.1245	92.5%	0.8988	0.104	88.4%	0.4873	0.0875	82.0%
64×10^{3}	1.0015	0.1292	87.1%	0.6237	0.0995	84.0%	0.3619	0.0884	75.6%
128×10^{3}	0.6982	0.1259	82.0%	0.4861	0.1049	78.4%	0.2937	0.0868	70.4%
256×10^{3}	0.5848	0.1298	77.8%	0.3733	0.1316	64.7%	0.2627	0.0998	62.0%

Note: "C" represents control wells, "I" represents inhibition wells, and "IR" represents inhibition rate. The inhibitory concentration of CPPU was 100 ng/mL.

Table S6. The checkered ic-ELISA result of purified antibody 9G9.

**Dilution of immunogen**	**2×10³**			**4×10³**			**16×10³**		
**(Serum dilution)**	**C**	**I**	**IR**	**C**	**I**	**IR**	**C**	**I**	**IR**
4×10³	3.9477	0.3176	92.0%	Overflow	0.1794	-	3.7747	0.1076	97.1%
8×10³	3.9192	0.1967	95.0%	3.9615	0.1185	97.0%	3.4797	0.1262	96.4%
16×10³	2.9608	0.1251	95.8%	2.8022	0.0849	97.0%	2.3749	0.0894	96.2%
32×10³	2.0751	0.1113	94.6%	1.7566	0.0891	94.9%	1.4702	0.1223	91.7%
64×10³	1.1081	0.1172	89.4%	0.9183	0.0996	89.2%	0.9132	0.1074	88.2%
128×10³	0.6657	0.123	81.5%	0.7233	0.1141	84.2%	0.4674	0.1302	72.1%
256×10³	0.4353	0.1101	74.7%	0.1934	0.1095	43.4%	0.3242	0.115	64.5%
512×10³	0.3343	0.1182	64.6%	0.2908	0.1159	60.1%	0.2515	0.1291	48.7%
Dilution of immunogen	32×10³			64×10³			128×10³		
(Serum dilution)	C	I	IR	C	I	IR	C	I	IR
4×10³	2.9644	0.1212	95.9%	1.943	0.1412	92.7%	1.1993	0.1301	89.2%
8×10³	1.9627	0.1323	93.3%	1.4608	0.1371	90.6%	0.9904	0.1349	86.4%
16×10³	1.6284	0.109	93.3%	1.0596	0.1116	89.5%	0.6632	0.1091	83.5%
32×10³	0.9642	0.1115	88.4%	0.6312	0.1266	79.9%	0.4488	0.116	74.2%
64×10³	0.5638	0.0971	82.8%	0.3745	0.0967	74.2%	0.3016	0.1118	62.9%
128×10³	0.3702	0.1132	69.4%	0.2598	0.1395	46.3%	0.197	0.1241	37.0%
256×10³	0.2436	0.0993	59.2%	0.194	0.1386	28.6%	0.1673	0.1272	24.0%
512×10³	0.1895	0.1172	38.2%	0.1765	0.1423	19.4%	0.1718	0.1747	-1.7%

Note: “C” represents control wells, “I” represents inhibition wells, and “IR” represents inhibition rate. The inhibitory concentration of CPPU was 100 ng/mL.

Table S7. The checkered ic-ELISA result of 9A10.

**Dilution of immunogen**	**2×10³**			**4×10³**			**16×10³**		
**(Serum dilution)**	**C**	**I**	**IR**	**C**	**I**	**IR**	**C**	**I**	**IR**
4×10³	0.8473	0.1616	81%	0.4811	0.1756	64%	0.2017	0.1584	21%
8×10³	0.3881	0.1381	64%	0.318	0.1574	51%	0.1781	0.1517	15%
16×10³	0.3831	0.1403	63%	0.2382	0.1207	49%	0.1389	0.1173	16%
32×10³	0.2423	0.2726	-13%	0.3528	0.1227	65%	0.1418	0.1091	23%
64×10³	0.1832	0.1349	26%	0.1301	0.1179	9%	0.1264	0.1222	3%
128×10³	0.1644	0.1075	35%	0.1321	0.1294	2%	0.1684	0.16	5%
256×10³	0.1885	0.1652	12%	0.175	0.1748	0%	0.1439	0.1196	17%
512×10³	0.1984	0.1593	20%	0.154	0.1397	9%	0.1706	0.1382	19%
Dilution of immunogen	32×10³			64×10³			128×10³		
(Serum dilution)	C	I	IR	C	I	IR	C	I	IR
4×10³	0.2017	0.1584	21%	0.2752	0.2735	1%	0.3102	0.3015	3%
8×10³	0.1781	0.1517	15%	0.1348	0.1984	-47%	0.2663	0.3422	-29%
16×10³	0.1389	0.1173	16%	0.1295	0.1256	3%	0.1717	0.2884	-68%
32×10³	0.1418	0.1091	23%	0.1527	0.1328	13%	0.2495	0.2407	4%
64×10³	0.1264	0.1222	3%	0.1314	0.1751	-33%	0.1744	0.2062	-18%
128×10³	0.1684	0.16	5%	0.2219	0.1937	13%	0.1784	0.1916	-7%
256×10³	0.1439	0.1196	17%	0.179	0.1858	-4%	0.2126	0.2595	-22%
512×10³	0.1706	0.1382	19%	0.2524	0.2655	-5%	0.2579	0.2221	14%

Note: "C" represents control wells, "I" represents inhibition wells, and "IR" represents inhibition rate. The inhibitory concentration of CPPU was 100 ng/mL.

Table S8. The checkered ic-ELISA result of 1B6.

**Dilution of immunogen**	**2×10³**			**4×10³**			**16×10³**		
**(Serum dilution)**	C	I	IR	C	I	IR	C	I	IR
4×10³	3.6744	0.4424	88.0%	3.142	0.2573	91.8%	1.5635	0.1544	90.1%
8×10³	2.5093	0.2898	88.5%	2.3126	0.1773	92.3%	1.2	0.1579	86.8%
16×10³	1.5815	0.1852	88.3%	1.3101	0.1238	90.6%	0.7002	0.1264	81.9%
32×10³	1.0341	0.1436	86.1%	0.7535	0.111	85.3%	0.4285	0.1375	67.9%
64×10³	0.5331	0.0926	82.6%	0.456	0.0823	82.0%	0.2991	0.1149	61.6%
128×10³	0.3113	0.0811	73.9%	0.2672	0.0757	71.7%	0.177	0.1231	30.5%
256×10³	0.2285	0.0792	65.3%	0.2156	0.0748	65.3%	0.1702	0.1355	20.4%
512×10³	0.1397	0.0793	43.2%	0.1353	0.077	43.1%	0.1338	0.1353	-1.1%
Dilution of immunogen	32×10³			64×10³			128×10³		
(Serum dilution)	C	I	IR	C	I	IR	C	I	IR
4×10³	1.27	0.1335	89.5%	0.7361	0.1654	77.5%	0.6426	0.2429	62.2%
8×10³	0.7881	0.1227	84.4%	0.5176	0.1298	74.9%	0.4248	0.1969	53.6%
16×10³	0.4596	0.1333	71.0%	0.3359	0.1577	53.1%	0.2832	0.1709	39.7%
32×10³	0.2739	0.1235	54.9%	0.2165	0.1215	43.9%	0.2316	0.2652	-14.5%
64×10³	0.2365	0.1151	51.3%	0.1783	0.1305	26.8%	0.2284	0.1906	16.5%
128×10³	0.153	0.1241	18.9%	0.1557	0.1237	20.6%	0.1622	0.1651	-1.8%
256×10³	0.1779	0.1159	34.9%	0.1533	0.1538	-0.3%	0.1956	0.1837	6.1%
512×10³	0.1314	0.1226	6.7%	0.1451	0.1547	-6.6%	0.1848	0.1758	4.9%

Note: "C" represents control wells, "I" represents inhibition wells, and "IR" represents inhibition rate. The inhibitory concentration of CPPU was 100 ng/mL.

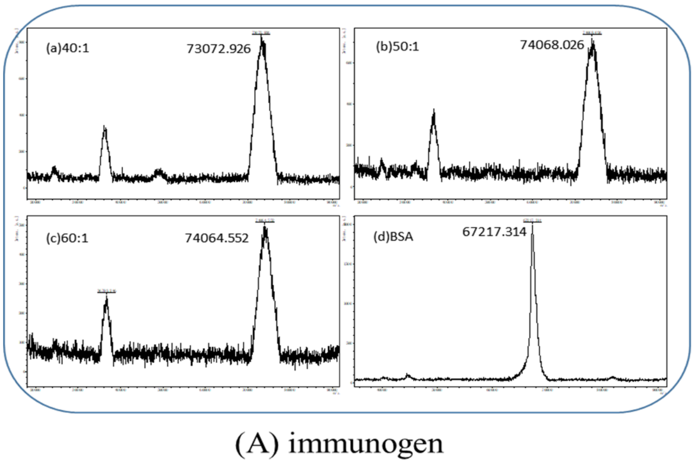

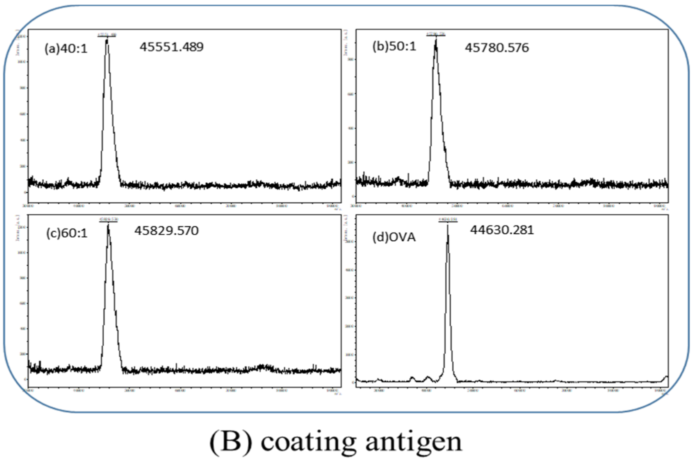

Figure S1. MALDI-TOF-MS results of the immunogen (A) and coating antigen (B).

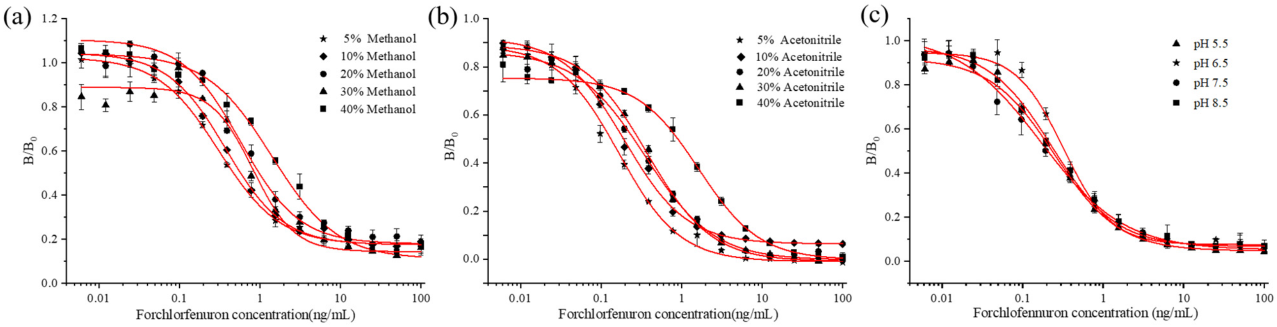

Figure S2. Optimization of ic-ELISA with different organic solvent contents and pH values in PBSTG buffer: (a) methanol, (b) acetonitrile, and (c) pH.

Table S9. Optimization of ic-ELISA with different organic solvent contents in PBSTG buffer.

**Methanol content**	**5%**	**10%**	**20%**	**30%**	**40%**
IC_{50}	0.47	0.58	0.82	0.98	1.59
IC_{80}	6.42	6.84	7.77	4.40	9.41
IC_{20}	0.13	0.18	0.23	0.37	0.34
R^2 of the calibration curve	0.998	0.998	0.996	0.994	0.997
Acetonitrile content	5%	10%	20%	30%	40%
IC_{50}	0.20	0.19	0.40	0.44	1.76
IC_{80}	0.93	0.86	2.50	2.28	9.74
IC_{20}	0.05	0.04	0.08	0.11	0.38
R^2 of the calibration curve	0.998	0.999	0.997	0.994	0.999

Table S10. Optimization of ic-ELISA with different pH values of PBSTG buffer.

**pH**	**5.5**	**6.5**	**7.5**	**8.5**
IC_{50}	0.25	0.33	0.21	0.28
IC_{80}	1.14	1.08	1.30	1.23
IC_{20}	0.06	0.11	0.04	0.08
R^2 of the calibration curve	0.998	0.997	0.998	0.998

Table S11. LC–MS/MS detection conditions.

**Liquid Phase condition**		
**Column**	**ACQUITY UPLC®BEH C18 column (100 mm × 2.1 mm., 1.7 μm)**	
Column Temperature	40 ℃	
Inject Volume	5 μL	
Mobile Phases Flow	0.3 mL/min	
Mobile Phases Consist Time(min)	A (0.1% Formic acid-H_2O)	B (0.01 M Ammonium formate-Methanol)
0	10	90
1.5	90	10
2.5	90	10
2.6	10	90
6	10	90
Mass Spectrum condition		
Monitoring Ion and Pairs/Collision Energy	248/129*<br>248/155	30eV<br>17eV
Scanning Mode	Multiple Reaction Monitoring (MRM)	
Dwell Time	100 ms	
Ion Mode	ESI+	
Nebulizing Gas	N_2	

Nebulizing Gas Flow	3 L/min
Heating Gas	N_2
Heating Gas Flow	10 L/min
DL Temperature	250℃
Heat Block Temperature	400℃
Drying Gas Flow	10 L/min

Note: *Quantitative ion

Table S12. Anti-CPPU 9G9 mAb type.

Antibody type	IgG1	IgA	IgG2a	IgG2b	IgG3	IgM
OD_{450nm}	1.7198	0.8808	0.8723	0.8317	0.8085	0.9189

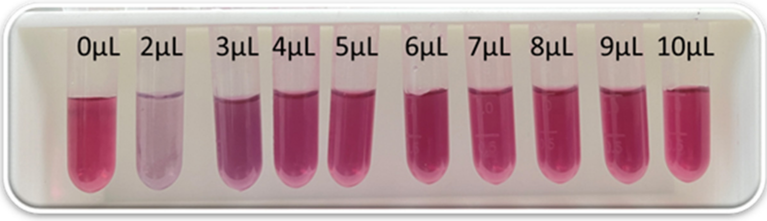

Figure S3. pH optimization of colloidal labeled antibody (the volume of K_2CO_3).

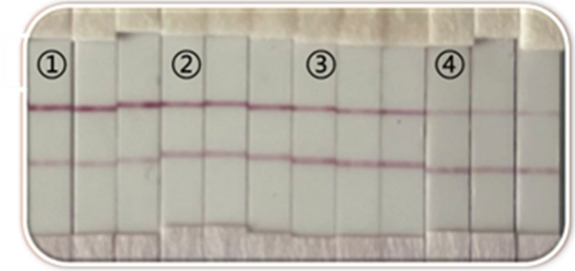

Figure S4. Optimization of coating antigen and second antibody concentration. (coating antigen/secondary antibody: ①2and1 mg/mL, ②1.5 and 0.8 mg/mL, ③3 and 0.4 mg/mL, ④4 and 0.2 mg/mL)

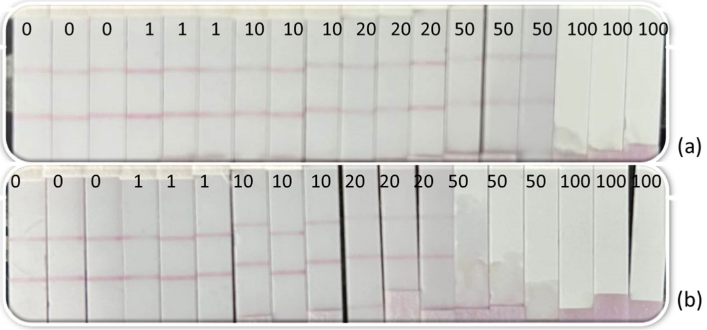

Figure S5. Organic solvent content on the strip: (a) methanol and (b) acetonitrile.

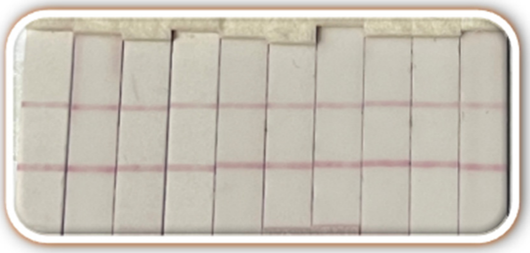

(a)

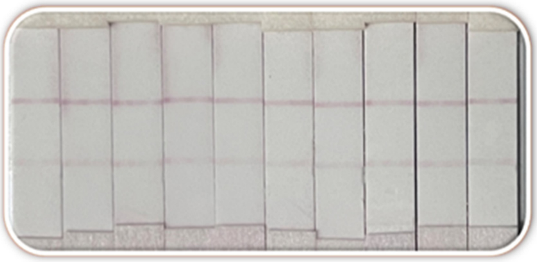

(b)

Figure S6. Test stability of CGN-ICTS (a) CK (b) 50 ng/mL.
